# Multiple Reaction Monitoring-Based Targeted Assays for the Validation of Protein Biomarkers in Brain Tumors

**DOI:** 10.3389/fonc.2021.548243

**Published:** 2021-05-14

**Authors:** Saicharan Ghantasala, Medha Gayathri J. Pai, Deeptarup Biswas, Nikita Gahoi, Shuvolina Mukherjee, Manubhai KP, Mehar Un Nissa, Alisha Srivastava, Sridhar Epari, Prakash Shetty, Aliasgar Moiyadi, Sanjeeva Srivastava

**Affiliations:** ^1^ Centre for Research in Nanotechnology and Science, Indian Institute of Technology Bombay, Mumbai, India; ^2^ Department of Biosciences and Bioengineering, Indian Institute of Technology Bombay, Mumbai, India; ^3^ Department of Genetics, University of Delhi, New Delhi, India; ^4^ Department of Pathology, Tata Memorial Centre’s – Advanced Centre for Treatment, Research and Education in Cancer, Navi Mumbai, India; ^5^ Homi Bhabha National Institute, Mumbai, India; ^6^ Department of Neurosurgery, Tata Memorial Centre’s – Advanced Centre for Treatment, Research and Education in Cancer, Navi Mumbai, India

**Keywords:** targeted proteomics, gliomas, Meningioma, Medulloblastoma, multiple reaction monitoring

## Abstract

The emergence of omics technologies over the last decade has helped in advancement of research and our understanding of complex diseases like brain cancers. However, barring genomics, no other omics technology has been able to find utility in clinical settings. The recent advancements in mass spectrometry instrumentation have resulted in proteomics technologies becoming more sensitive and reliable. Targeted proteomics, a relatively new branch of mass spectrometry-based proteomics has shown immense potential in addressing the shortcomings of the standard molecular biology-based techniques like Western blotting and Immunohistochemistry. In this study we demonstrate the utility of Multiple reaction monitoring (MRM), a targeted proteomics approach, in quantifying peptides from proteins like Apolipoprotein A1 (APOA1), Apolipoprotein E (APOE), Prostaglandin H2 D-Isomerase (PTGDS), Vitronectin (VTN) and Complement C3 (C3) in cerebrospinal fluid (CSF) collected from Glioma and Meningioma patients. Additionally, we also report transitions for peptides from proteins – Vimentin (VIM), Cystatin-C (CST3) and Clusterin (CLU) in surgically resected Meningioma tissues; Annexin A1 (ANXA1), Superoxide dismutase (SOD2) and VIM in surgically resected Glioma tissues; and Microtubule associated protein-2 (MAP-2), Splicing factor 3B subunit 2 (SF3B2) and VIM in surgically resected Medulloblastoma tissues. To our knowledge, this is the first study reporting the use of MRM to validate proteins from three types of brain malignancies and two different bio-specimens. Future studies involving a large cohort of samples aimed at accurately detecting and quantifying peptides of proteins with roles in brain malignancies could potentially result in a panel of proteins showing ability to classify and grade tumors. Successful application of these techniques could ultimately offer alternative strategies with increased accuracy, sensitivity and lower turnaround time making them translatable to the clinics.

## Introduction

Advances in Mass spectrometry have provided a major impetus to the field of proteomics over the last decade. These developments have resulted in proteomics playing a role in advancing our understanding of disease biology and opened new avenues such as biomarker development, augmentation of therapeutic modalities and drug discovery. Global consortia like CPTAC (Clinical Proteomics Tumor Analysis Consortium), TCGA (The Cancer Genome Atlas) and HUPO (Human Proteome Organization) have played a major role in utilising the power of omics technologies towards understanding the underlying mechanisms of various cancers (1–3). The development of new softwares, global repositories and reproducible workflows has also played a key role in increasing the utility of proteomics methodologies for research in the last decade. However, despite these advancements in technology and our understanding of cancer, early detection and treatment of malignancies related to the brain continue to pose a serious challenge even today (4). Primary brain tumors are a heterogeneous group of malignant and benign tumors arising from brain parenchyma or the cell types existing in the cranial vault including cranial nerves, meninges, neuroepithelial tissues, germ cells, pituitary gland, and even residual embryonic tissues. These tumors are characterized by high morbidity and mortality rates due to their localization, invasive growth and heterogeneous nature (5). Among all CNS tumors, glioma accounts for approximately 28% of tumors, of which 80% are malignant (6). Glioblastomas are the most prevalent and malignant sub-type of gliomas (3.20 per 100,000 population) followed by diffuse astrocytomas (0.48 per 100,000 population) (7). Meningiomas are slow-growing extra-axial lesions, originating from cells of arachnoid villi or meningothelial cells present in the meninges. These are most prevalent primary brain tumors and their incidences seem to rise with an increase in age. Medulloblastomas are embryonal tumors commonly seen in children. All medulloblastomas are classified pathologically as Grade IV due to their aggressive nature. Transcriptomic studies have identified four subtypes of medulloblastoma, which include SHH, WNT, G4 and G3, each with a distinct clinical and therapeutic presentation (8). The 2016 classification of WHO for brain cancers included molecular markers along with histological parameters for better clinical identification (9).

Recent advances in molecular biology and genomics have immensely benefitted the classification and prognosis of brain cancers. The omics research has also contributed in the brain tumour investigation which has unravelled different underlying biological mechanism, accelerated biomarker discovery (10–12). However, there still exits a gap between direct implementation of these studies in the clinics, as brain tumors are among the deadliest cancers. Successful translation of candidate biomarkers is also limited due to the unavailability of antibodies required for the validation experiments on large cohorts. The recognition of the potential of targeted proteomics approaches such as selected reaction monitoring, accelerated its use in hypothesis driven proteomics research (13). These proteomics approaches offer advantages such as high sensitivity and accuracy over the conventional validation approaches which rely on the use of antibodies, are labour intensive and time consuming. These approaches are slowly but surely finding use in biomedical research (14). Faria et al. has extensively reviewed the revolutionary role of targeted proteomics in cancer biomarker discovery and its shift from shotgun proteomics (15). Targeted proteomics has also showed its immense potential in the field of infectious disease, understanding metabolic disorders and in accelerating our understanding of the SARS CoV2 virus and its mode of infection during the ongoing pandemic (16–18). The integration of proteogenomics and targeted proteomic validation could be a promising tool owing to increased robustness, sensitivity and selectivity in cancer research (19). The Verification Working Group (VWG) of the CPTAC consortium carried out multiple experiment with an aim to assess the reproducibility, robustness, and transferability of MRM based assays. The findings from the study highlight the utility and practicality of highly reproducible MS-based assays and their potential role in clinics (20). In the current study, we have employed MRM as a proof of concept to validate a few proteins previously identified in our discovery studies on the three major brain malignancies - Gliomas, Meningiomas and Medulloblastomas. To our knowledge, this is the first study to validate potential biomarkers in brain tumors from CSF and tissue proteins using mass spectrometry-based MRM approach.

## Materials and Methods

### Preparation of the Clinical Samples for Mass Spectrometric Analysis

#### Glioma and Meningioma CSF

Protein extraction from 17 CSF samples (MG I (n=3), MG II (n=4), GBM (n=5) and LGG or low-grade gliomas (n=5)) from Glioma and Meningioma patients was performed using the urea lysis protocol optimized in our lab. Please refer to **Supplementary Table 1** for clinical information about the samples used. 500µL of CSF from Meningioma and Glioma patients was concentrated to 200µL each and processed. The CSF samples were sonicated at an amplitude of 20% with 5 sec pulse for a total of 8 cycles. Following this, the samples were vacuum dried, and the pellet was reconstituted in 6M Urea buffer. Protein amount in the sample was then quantified using 2D quant kit (GE-Healthcare, Sweden). From this, 50µg of protein sample was reduced with 0.5M TCEP at a final concentration of 20mM for one hour at 37°C followed by alkylation using 37.5mM IAA for 30 minutes at RT. The samples were diluted with 25mM Tris buffer (pH 8.0) to reduce the urea concentration to less than 1M. Protein digestion was carried out by adding Trypsin (Pierce) to the tubes in the ratio of 1:30 followed by incubation overnight at 37°C. After digestion, the contents of the tube were dried in a vacuum concentrator, reconstituted in 0.1% FA, and desalted using C18 stage tips. Finally, the desalted peptides were stored at -80°C for further use.

#### Meningioma, Glioma and Medulloblastoma Tissue

Surgically resected tissue samples from meningioma, glioma and medulloblastoma patients were obtained from Tata Memorial Hospital, Mumbai. Representative MRI images for Meningioma, Glioma and Medulloblastoma can be found in **Figures 2A** and **4A**, respectively. All the relevant clinical information can be found in **Supplementary Table 1**. In brief, the tissue samples were flash frozen in liquid nitrogen and stored at -80°C. The protein extraction was done using Urea buffer (8M Urea, Tris-HCl buffer) with addition of Phosphatase inhibitor cocktail (Sigma Aldrich^®^) (21). Furthermore, the reduced and alkylated proteins were digested by addition of Trypsin (Pierce, Thermofisher Scientific) followed by overnight incubation at 37°C. The digested peptides were then vacuum dried and desalted using *in-house* C18 stage tips. The tissue sample set had Meningioma (n=6), Glioma (n=6) and Medulloblastoma (n=6) in addition to 3 control tissues for each tumor, arachnoid tissue (n=3) for Meningioma, peritumoral tissue (n=3) for Glioma and cerebellum tissue (n=3) for Medulloblastoma.

### Liquid Chromatography and Triple Quadrupole Parameters

All the MRM experiments were carried out on TSQ Altis mass spectrometer (ThermoFisher Scientific, USA) coupled to a Vanquish uHPLC (ThermoFisher Scientific, USA) platform. 1µg of peptides was loaded on to a Hypersil Gold C18 column 1.9μm 100 X 2.1mm (ThermoFisher Scientific, USA) and chromatographic separation of peptides was carried out at a flow rate of 0.40ml/min for CSF samples and 0.45ml/min for tissue samples. The total time of gradient for CSF samples was 20 minutes, while that for tissue samples was 10 minutes. The buffer system was binary with Buffer A (0.1% Formic acid in water) and Buffer B (80% Acetonitrile in 0.1% Formic acid water). The gradients used have been shown in **Supplementary Table 2**. With an ESI source to the MS, the data was acquired for 20 minutes in case of CSF samples and 10 minutes in case of tissue samples. All the other MS relevant parameters (which were kept similar for both kinds of samples) are tabulated in **Supplementary Figure 1**.

### BSA and MCF-7 as Quality Check Standards

To monitor the instrument behaviour and consistency in response, peptides from Bovine serum albumin (BSA) and MCF-7 cell line pellets were prepared with the urea extraction method as explained above for tissue samples. The transition lists were optimised and a final method containing 7 peptides DLGEEHFK, LVNELTEFAK, DDSPDLPK, AEFVEVTK, HLVDEPQNLIK, LGEYGFQNALIVR and QTALVELLK was prepared. Similarly, the refined list of MCF-7 had 2 proteins namely Fructose Bisphosphate Aldolase or ALDOA (GILAADESTGSIAK and ADDGRPFPQVIK) and Glyceraldehyde-3-phosphate dehydrogenase or GAPDH (LVINGNPITIFQER, GALQNIIPASTGAAK, VIPELNGK and LISWYDNEFGYSNR). To ensure the quantitative linearity of the instrument, we also injected 200ng, 400ng, 600ng, 800ng and 1µg of BSA. The results were imported in Skyline and peak areas acquired were plotted to check for linearity in the quantitative values.

### Transition List Preparation and Data Analysis

**Figure 1:**
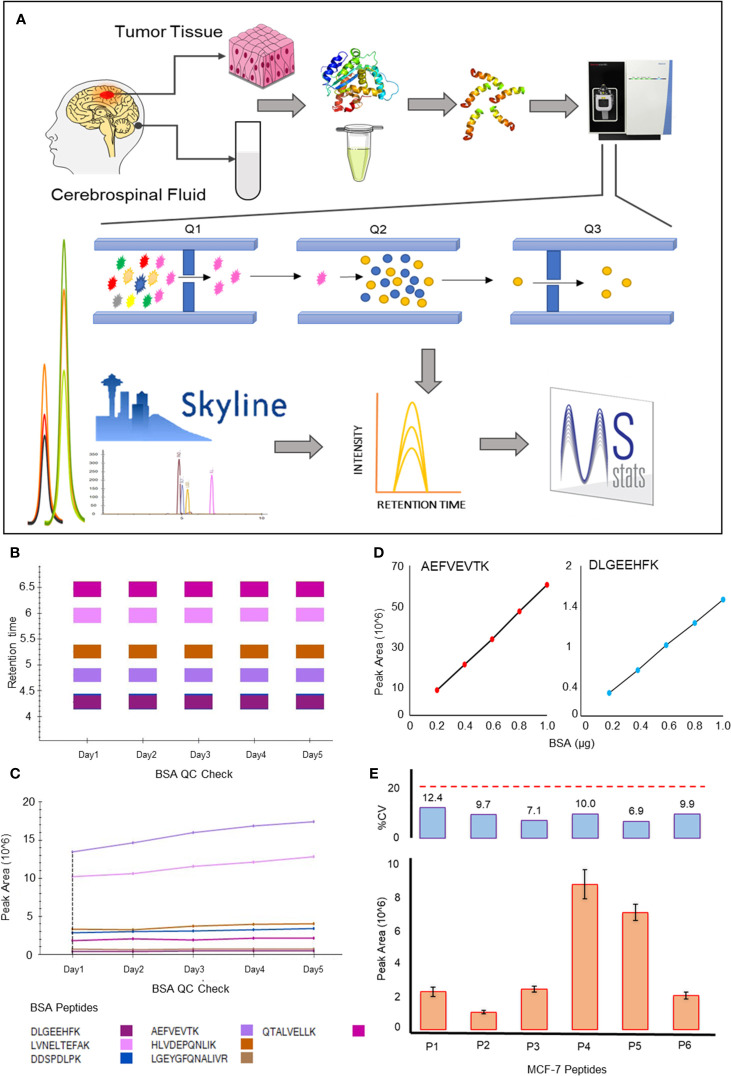
Schematic of the MRM workflow and QC. **(A)** Schematic outline of the MRM based experiments from biological specimens; **(B, C)** Response of seven peptides of BSA (DLGEEHFK, LVNELTEFAK, DDSPDLPK, AEFVEVTK, HLVDEPQNLIK, LGEYGFQNALIVR, QTALVELLK) monitored during the experiments in terms of Retention time and Peak area respectively; **(D)**. Quantification sensitivity of the instrument using peak area against concentration (in µg) curve of two peptides of BSA injected in the increasing concentration; **(E)** shows the repeatability and variation in the response of six peptides belongs to two proteins of MCF-7 digested peptide used as a QC for the experiments (P1: GILAADESTGSIAK and P2: ADDGRPFPQVIK of ALDOA whereas P3: LVINGNPITIFQER, P4: GALQNIIPASTGAAK, P5: VIPELNGK and P6: LISWYDNEFGYSNR belongs to GAPDH).

Proteins and their peptides for MRM experiments were selected based on data from shotgun experiments and information available in SRM Atlas (22). The peptide sequences were imported into Skyline and transition list created with peptides having 8-20 amino acids (23). All the selected peptides were unique with 0 missed cleavage. The transition list included y-ions from “ion 3” to “last ion -3”. Method files were created for the unrefined transition list for the selected proteins and initial optimization was carried out to select the best peptides and their transitions for each protein using the sample pool. After screening the transitions from the first round of experiments, a final transition file with refined transitions was prepared. Finally, the transitions of each tumour type were monitored for each sample using cycle time of 2 second and resolution of 0.7 m/z for both Q1 and Q3. The data obtained was further analysed using Skyline daily version 20.2.1.384. The fold changes were calculated using the MSstats tool in Skyline keeping a confidence interval of 95% (24). The schematic workflow of the experiment has been shown in **Figure 1A**.

## Results

### Quality Check Using BSA and MCF-7

We performed quality checks at the following levels: 1) BSA: A single injection of 300ng of BSA was done every day when the samples were run on the instrument. The refined transition list containing 7 peptides for BSA gave uniform response. A representation of this can be seen in **Figures 1B, C**. The peak areas for BSA were found to be similar on all the five consecutive days the sample was injected. 2) The instrument response was found to be linear when increasing concentrations of BSA were injected (**Figure 1D**). 3) The CV values calculated using peak area values for 500ng of MCF-7 injected on five consecutive days were observed to be less than 15% (**Figure 1E**). These quality check steps helped us decipher the repeatability, reproducibility and efficiency of the instrument.

### Monitoring of Potential Protein Markers in Meningioma and Glioma CSF

**Figure 2:**
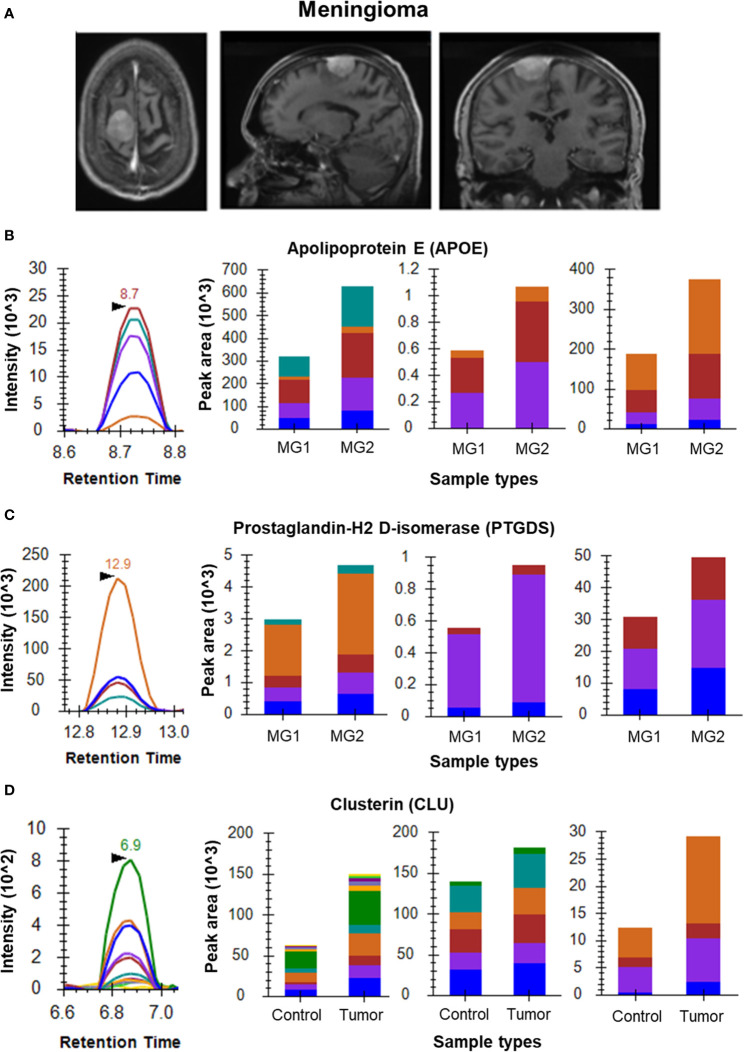
MRM analysis of Meningioma CSF and tissue samples. **(A)** Radiological images of Meningioma – contrast MRI images (axial, sagittal and coronal views, respectively); **(B)** Representative peak shape for AATVGSLAGQPLQER and bar plots of AATVGSLAGQPLQER, LGPLVEQGR and SELEEQLTPVAEETR, respectively showing overexpression of APOE in Grade II meningioma (n=4) as compared to Grade I meningioma (n=3) in CSF samples; **(C)** Representative peak shape for AQGFTEDTIVFLPQTDK and bar plots of AQGFTEDTIVFLPQTDK, TMLLQPAGSLGSYSYR and WFSAGLASNSSWLR, respectively showing overexpression of PTGDS in Grade II meningioma as compared to Grade I meningioma in CSF samples; **(D)** Representative MRM peak for one peptide of CLU and bar plots depicting the increased expression of peptides EILSVDCSTNNPSQAK, ELDESLQVAER and LFDSDPITVTVPVEVSR of CLU in Meningioma tumor tissue samples (n=6) as compared to arachnoid controls (n=3).

**Figure 3:**
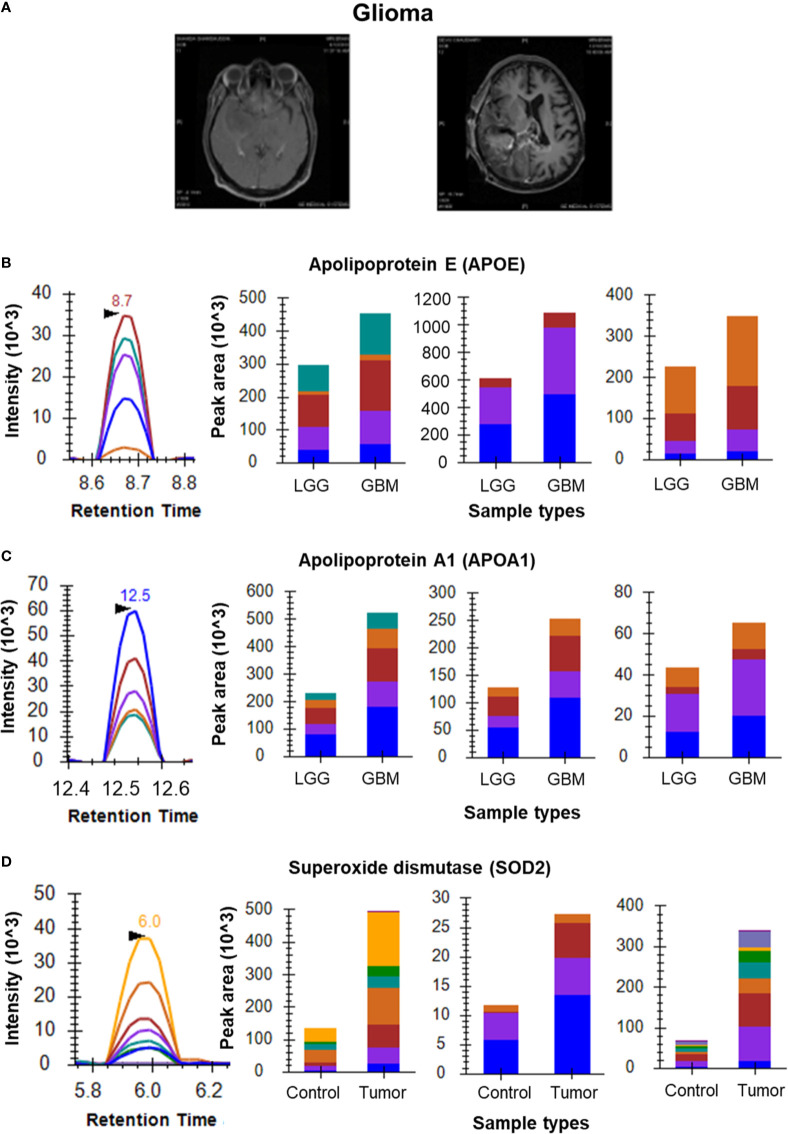
MRM analysis of Glioma CSF and tissue samples. **(A)** T1 contrast images showing Low Grade Glioma and High Grade Glioma, respectively; **(B)** Representative peak shape for AATVGSLAGQPLQER and bar plots of AATVGSLAGQPLQER, LGPLVEQGR and SELEEQLTPVAEETR, respectively showing overexpression of APOE in CSF samples of GBM (n=5) as compared to LGG (n=5); **(C)** Representative peak shape for LLDNWDSVTSTFSK and bar plots of LLDNWDSVTSTFSK, DYVSQFEGSALGK and ATEHLSTLSEK, respectively showing overexpression of APOA1 in GBM CSF samples as compared to LGG CSF samples; **(D)** Representative MRM peak for one peptide of SOD2 and bar plots depicting the increased expression of peptides GDVTAQIALQPALK, LLDNWDSVTSTFSK and ATEHLSTLSEK of SOD2 in Glioma tumor tissue samples (n=6) as compared to peritumoral controls (n=3).

Initial optimization experiments were performed to select the best flying peptides and their transitions using pooled samples. Three peptides of 5 Proteins which includes APOA1, APOE, PTGDS, VTN and C3 were monitored for both Meningioma and Glioma samples. For the meningioma CSF samples, three peptides of APOE (SELEEQLTPVAEETR, LGPLVEQGR and AATVGSLAGQPLQER) gave a cumulative fold change of 2.21 whereas peptides of PTGDS (WFSAGLASNSSWLR, TMLLQPAGSLGSYSYR and AQGFTEDTIVFLPQTDK) showed a fold change of 1.52 (**Figures 2B, C**). However, the cumulative fold change of APOA1 (DYVSQFEGSALGK, LLDNWDSVTSTFSK and ATEHLSTLSEK), VTN (DVWGIEGPIDAAFTR, FEDGVLDPDYPR and SIAQYWLGCPAPGHL) and C3 (LVAYYTLIGASGQR, TGLQEVEVK and SGSDEVQVGQQR) showed upregulation in Grade I in comparison to Grade II (**Supplementary Figure 2**). For Glioma, 3 peptides each of the above mentioned 5 proteins were chosen to look for differential expression between the grades. The same three peptides of APOE and APOA1 gave a cumulative fold change of 1.59 and 2.69 respectively in GBM when compared to low grade glioma (**Figures 3B, C**). While cumulative fold change of three peptides of VTN, PTGDS and C3 in GBM were found to be upregulated in GBM as compared to low grade glioma (**Supplementary Figure 2**). A final list of these proteins and peptides has been provided in **Supplementary Table 3**.

### Monitoring of Potential Biomarkers From Tumor Tissue Specimens

Differential Protein expression for a few candidate biomarkers was observed for by comparing their expression in brain tumor tissues to that in their respective control tissues. For the meningioma tissue samples, we observed overexpression of VIM, CST3 and CLU. The three selected peptides of CLU (EILSVDCSTNNPSQAK, ELDESLQVAER and LFDSDPITVTVPVEVSR) gave a cumulative fold change of 1.41 (**Figure 2D**). While the three peptides of CST3, namely LVGGPMDASVEEEGVR, QIVAGVNYFLDVELGR and TQPNLDNCPFHDQPHLK gave a cumulative fold change of 1.53. Four peptides of VIM (SLYASSPGGVYATR, ILLAELEQLK, LGDLYEEEMR and FADLSEAANR) showed a cumulative fold change of 2.25 as compared to the arachnoid controls with a confidence interval of 95% (**Supplementary Figure 3**).

For Glioma tissue samples we screened several known biomarker proteins including ANXA1, SOD2 and VIM. Three peptides of SOD2 (GDVTAQIALQPALK, GELLEAIK and AIWNVINWENVTER) showed a cumulative positive fold change of 2.48 in Glioma samples as compared to the peritumoral controls (**Figure 3D**). ANXA1 was found to be upregulated in Glioma tissues (peptides GLGTDEDTLIEILASR, GVDEATIIDILTK and GTDVNVFNTILTTR). Apart from the above-mentioned peptides of VIM, we observed QVQSLTCEVDALK and ETNLDSLPLVDTHSK of VIM also gave good response for glioma samples. These 6 peptides showed overexpression in tumor tissue *vs* peritumoral tissue (**Supplementary Figure 3**). It is to be noted that one of the controls in this samples set, did not give good response and hence was excluded from the fold change calculations. The acquired data for this outlier has been provided in our data. Peritumoral tissues are rare to come by hence the analysis included only two controls.

**Figure 4:**
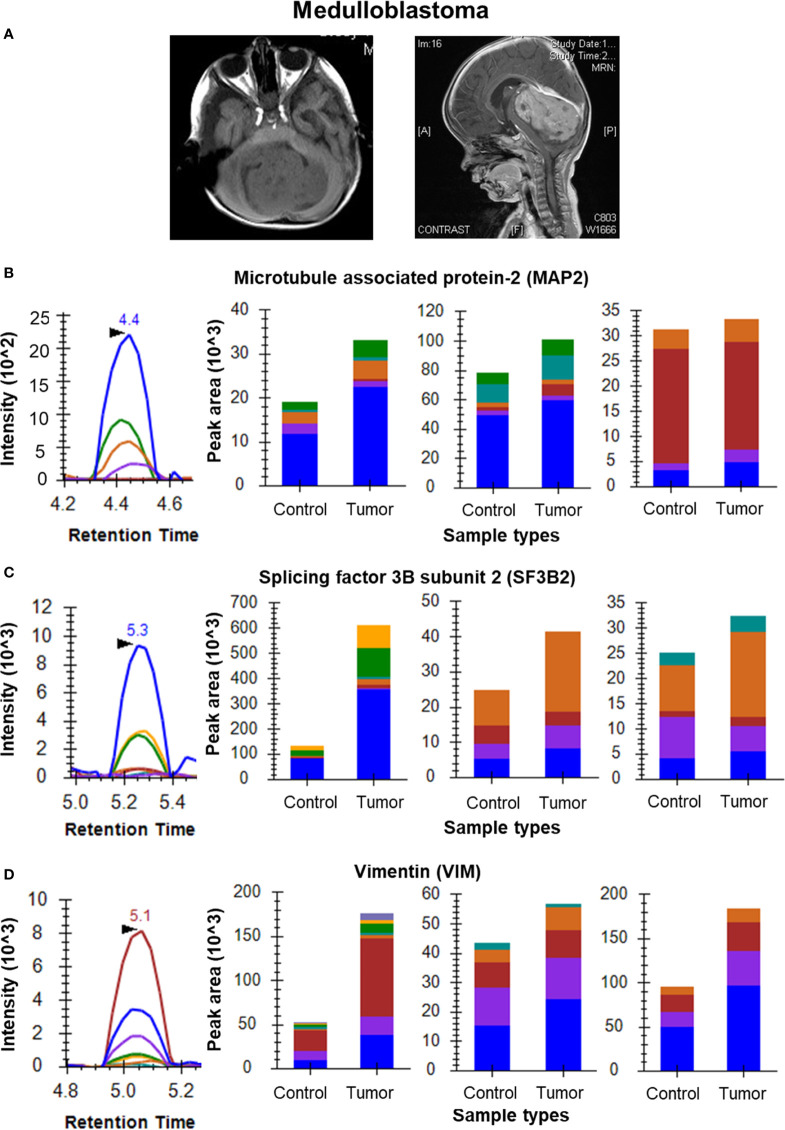
MRM analysis of Medulloblastoma tissue samples. **(A)** MRI images of a medulloblastoma showing a mass on T1 weighted image and the sagittal contrast image showing the extent of the tumor, respectively; **(B)** MRM peak shape for TPGTPGTPSYPR of MAP2 and bar plots of TPGTPGTPSYPR, VGSLDNAHHVPGGGNVK and VDHGAEIITQSPGR, respectively showing overexpression of MAP2 in tumor tissue (n=6) as compared to cerebellar controls (n=3); **(C)** MRM peak shape for VGEPVALSEEER of SF3B2 and bar plots of VGEPVALSEEER, KPGDLSDELR and YGPPPSYPNLK, respectively showing overexpression of SF3B2 in tumor tissue as compared to cerebellar controls; **(D)** MRM peak shape for SLYASSPGGVYATR of VIM and bar plots of SLYASSPGGVYATR, ILLAELEQLK and FADLSEAANR, respectively showing overexpression of VIM in tumor tissue as compared to cerebellar controls.

For the Medulloblastoma tissue sample set, the data clearly shows overexpression of MAP2 in tumor tissue with a fold change of 1.34. Peptides TPGTPGTPSYPR, VGSLDNAHHVPGGGNVK and VDHGAEIITQSPGR were monitored for MAP2 in the individual samples (**Figure 4B**). Three peptides of SF3B2 (VGEPVALSEEER, KPGDLSDELR and YGPPPSYPNLK) showed a cumulative fold change of 2.89 whereas the four peptides of VIM (SLYASSPGGVYATR, ILLAELEQLK, LGDLYEEEMR and FADLSEAANR) gave cumulative fold change of 2.39 (**Figures 4C, D**). The final list of all the selected proteins and their peptides for Glioma, Meningioma and Medulloblastoma has been provided in **Supplementary Table 4**.

## Discussion

Mass Spectrometry based targeted proteomics approaches like MRM require considerable optimization and investment of time. These methods are increasingly replacing the conventional molecular biology methods owing to their superior accuracy and reproducibility over techniques like western blotting and immunohistochemistry which rely heavily on the use of antibodies. In the current study we have reported the use of MRM to accurately detect and quantify proteins and their peptides from biospecimens CSF and tumor tissue in three brain malignancies. These proteins have been reported to play important roles in development of these brain tumors and accurate detection and quantification of such proteins can greatly advance our understanding of tumor pathobiology.

The expression levels of proteins such as VTN, APOA1 and PTGDS were found to be highly up regulated in GBM as compared to low grade gliomas. Extracellular matrix (ECM) remodelling is one of mechanisms involved in tumor maturation and migration. The ECM of adult brain is characterized by absence of most of the adhesive glycoproteins that aid in cell attachment and invasion (25). However, to promote cell attachment and migration, malignant astrocytomas remodel the ECM through synthesis of VTN (26). Our MRM data from CSF of gliomas also suggests a significant up-regulation of VTN in GBMs as compared to LGGs, one of the interactors of Integrins. In CSF of Meningiomas, the levels of VTN were found to be higher in Grade I as compared to Grade II. Integrins are the cell-surface heterodimeric receptors that integrate ECM with intracellular cytoskeleton to mediate cell adhesion, migration and invasion. Malignant astrocytomas are known to express integrins αvβ3 and αvβ5, which bind to VTN and RGD domain of osteopontin, thereby promoting integrin-mediated cell attachment and migration (26, 27).

The protein PTGDS has been reported as a potential biomarker of meningioma in multiple studies (28, 29). Kim et.al., reported that expression of PTGDS using Western blot was found to be decreased in Meningioma CSF when compared to non-tumor controls (29). The same protein has also been reported in glioblastoma using deep learning (30). Our study validated the higher expression of PTGDS in Grade I meningioma as compared to Grade II, however the expression of this protein was found to be upregulated in GBM in comparison to LGG. Another protein, Complement C3 (C3) has been reported to be key protein in tumorigenesis of Meningiomas (31). In our study, the protein was found to be down regulated in Meningioma Grade II when compared to Grade I. The levels of this protein in gliomas were observed to be opposite to that seen in Meningiomas with the high grade GBMs showing an up-regulation over the low grade gliomas.

Our discovery dataset and literature also pointed at up-regulation of several apolipoproteins in gliomas and meningiomas (12, 31, 32). APOE plays a vital role in redistribution of intracellular lipid and tissue reconstruction in CNS through a receptor dependent pathway. Astrocytes are one of the main sites of APOE synthesis (33). Nicoll et al., showed APOE immunoreactivity in the tumor cells, macrophages and nearby astrocytes, supporting the role of APOE in delivery of lipids to tumor cells and its recycling by macrophages in necrotic areas. Increased levels of lipids in serum of GBM patients and over expression of APOE in meningioma CSF sample in comparison to non-tumor CSF have also been reported (34) and (29). In the current study, we have observed an upregulation of APOE and APOA1 in GBMs when compared to LGGs and upregulation of APOE and downregulation of APOA1 in Meningioma Grade II tissue compared to Meningioma Grade I.

For tissue samples, the MRM experiments were performed on proteins with roles in tumor pathology curated from available literature. The Human Protein Atlas reports a higher expression of VIM in Gliomas (35, 36). Mukherjee et al. validated the expression of VIM in Meningiomas using MRM (37). In the current study, we have reported four peptides for VIM, not reported elsewhere further strengthening the claim for its use as a biomarker for meningiomas as reported by Mukherjee et al. In Meningiomas, the protein CLU was found to be upregulated when compared to controls. It has been reported as a meningioma associated marker in the literature and known to inhibit apoptosis (31, 38). We have also observed an up regulation of CST3 in meningioma tumour samples when compared to control tissues. CST3 is an inhibitor of cysteine proteases and has been reported to have a positive alteration in high-grade meningiomas (39).

Many studies have reported the role of ANXA1 and SOD2 in gliomas, by virtue of their increased expression levels in the tumor tissues (40). ANXA1 is known to alter regular cell proliferation, differentiation and works as a substrate of EGFR. We observed that the levels of peptides for ANXA1 and SOD2 were upregulated in glioma tissues in comparison to peritumoral control tissues.

MAP2 is a well-known neural marker for medulloblastoma as reported in a few studies (41–43). It is a frequently considered marker during IHC of MB samples. It has been observed in MB tumors irrespective of the age of the patient (44). Our data shows the overexpression of this protein in MB tumors as compared to controls. Cancerous mutations in splicing factor SF3B2 have been reported to affect the ubiquitinylation pathways and hence associated with cancer. SF3B2 has been reported as a potential gene marker in many diseases including medulloblastoma in DisGeNET and has appeared as one of the significantly dysregulated proteins in our discovery dataset (45). One of our recent studies on medulloblastoma has also highlighted the significance of splicing events in medulloblastoma disease biology (10). In our data, VIM was observed to be overexpressed in medulloblastoma as compared to controls in accordance with literature (12, 46, 47).

In conclusion, our study highlights the importance of targeted proteomics in detection and validation of proteins with roles in pathobiology of brain tumors. From our MRM experiments using CSF we report that APOE could be a potential tumor progression marker in Meningioma and Glioma. The trends for APOA1 were found to be opposite in Gliomas and Meningiomas, with higher expression in GBMs and Grade I meningiomas as compared to the LGGs and Grade II meningiomas, respectively. We also observed upregulation of VTN, PTGDS and APOA1 in CSF of GBM patients in comparison to CSF of LGG patients. The protein Vimentin was observed to be overexpressed in all the three brain tumor tissue samples. We have also validated tumor markers such as CLU and CST3 for Meningioma, ANXA1 and SOD2 in Glioma and MAP2 for Medulloblastoma. With the advancements in gene sequencing techniques, routine diagnosis for complex cancers has become easier, faster and efficient. However, there remains a greater reliance on the age-old molecular biology methods of Immunohistochemistry (IHC) and Fluorescence in-situ hybridization (FISH). In addition to the non-specific binding towards proteins, these antibody-based methods also have notoriously high chances of inter-observer variability leading to differences in grading the tumors (48). The MRM technique owing to its quantitative accuracy and sensitivity, can offer a suitable alternative to the more labour-intensive molecular biology techniques currently used. The development of an analytical method and assay based on MRM involves a multitude of aspects such as generating a calibration curve, determination of analytical specificity (selectivity or interference), analytical sensitivity, carryover, precision, recovery of assay, matrix effect, recovery of immunoprecipitation, dilution integrity, stability, reproducibility, and quality control (QC) of samples (49). Successful validation of proteins on a large cohort with easily obtainable biospecimens from patients can pave way to designing panels of protein markers with ability to distinguish between the grades of these tumors, thus providing a faster and more accurate alternative to the existing modalities.

## Data Availability Statement

The targeted proteomic datasets are deposited in the Panorama Public and can be accessed through this link: https://panoramaweb.org/Brain_Tumor_marker_MRM.url

## Ethics Statement

The studies involving Glioma patients was approved by the Institutional Review Board (IRB) at Tata Memorial Hospital (TMC-ACTREC Project No.15). The study on meningioma patients was approved as part of an institutional review board at TMH (TMC-ACTREC project no.149). For use of samples from patients with Medulloblastomas the approval was obtained from TMH IRB (TMC-ACTREC project no.197). The patients/participants provided their written informed consent to participate in this study.

## Author Contributions

SS, SG, MP, and DB planned the experiment. AM, and PS acquired clinical samples for the study. SE performed the grading and histopathological studies for the acquired clinical samples. DB, MP, SM, SG, NG, and MK carried out experiments. DB, MP, and SG compiled the data and wrote the manuscript. MN was involved in instrument setup and data acquisition. DB, MP, and AS performed statistical analysis. All authors contribted to the article and approved the submitted version.

## Funding

The study was funded through MHRD-UAY Project UCHHATAR AVISHKAR YOJANA; UAY-(MHRD), project #34_IITB (2016) to SS, MASSFIIT (Mass Spectrometry Facility, IIT Bombay; BT/PR13114/INF/22/206/2015) for MS-based proteomics experiments.

## Conflict of Interest

The authors declare that the research was conducted in the absence of any commercial or financial relationships that could be construed as a potential conflict of interest
