# Supplementary material for: Multiple Reaction Monitoring-Based Targeted Assays for the Validation of Protein Biomarkers in Brain Tumors
Source: Front Oncol. 2021 May 14;11:548243. doi: 10.3389/fonc.2021.548243 (PMC8162214; doi:10.3389/fonc.2021.548243)
Supplement: Supplementary file 1 [file Image_1.pdf]

Supplementary Figure 1

MS parameters

Method Summary

Method Settings

Method Duration (min): **10**

Global Parameters

Ion Source

Ion Source Type: **H-ESI**
Spray Voltage: **Static**
Positive Ion (V): **3500**
Negative Ion (V): **2500**
Current LC Flow (μL/min): **0**
Sheath Gas (Arb): **45**
Aux Gas (Arb): **10**
Sweep Gas (Arb): **1**
Ion Transfer Tube Temp (°C): **300**
Vaporizer Temp (°C): **350**
APPI Lamp: **Not in Use**

Experiment 1

Start Time (min): **0**
End Time (min): **10**

Master Scan:

SRM

Use Cycle Time: **True**
Cycle Time (sec): **2**
Use Calibrated RF Lens: **True**
Q1 Resolution (FWHM): **0.7**
Q3 Resolution (FWHM): **0.7**
CID Gas (mTorr): **2.5**
Source Fragmentation (V): **0**
Chromatographic Peak Width (sec): **15**
Use Chromatographic Filter: **False**
Use Retention Time Reference: **False**
Display Retention Time: **False**
Use Quan Ion: **False**
Show Visualization: **False**

SRM Table
