# Supplementary material for: Multiple Reaction Monitoring-Based Targeted Assays for the Validation of Protein Biomarkers in Brain Tumors
Source: Front Oncol. 2021 May 14;11:548243. doi: 10.3389/fonc.2021.548243 (PMC8162214; doi:10.3389/fonc.2021.548243)
Supplement: Supplementary file 2 [file Image_2.pdf]

Supplementary Figure 2A

Vitronectin (Meningioma CSF)

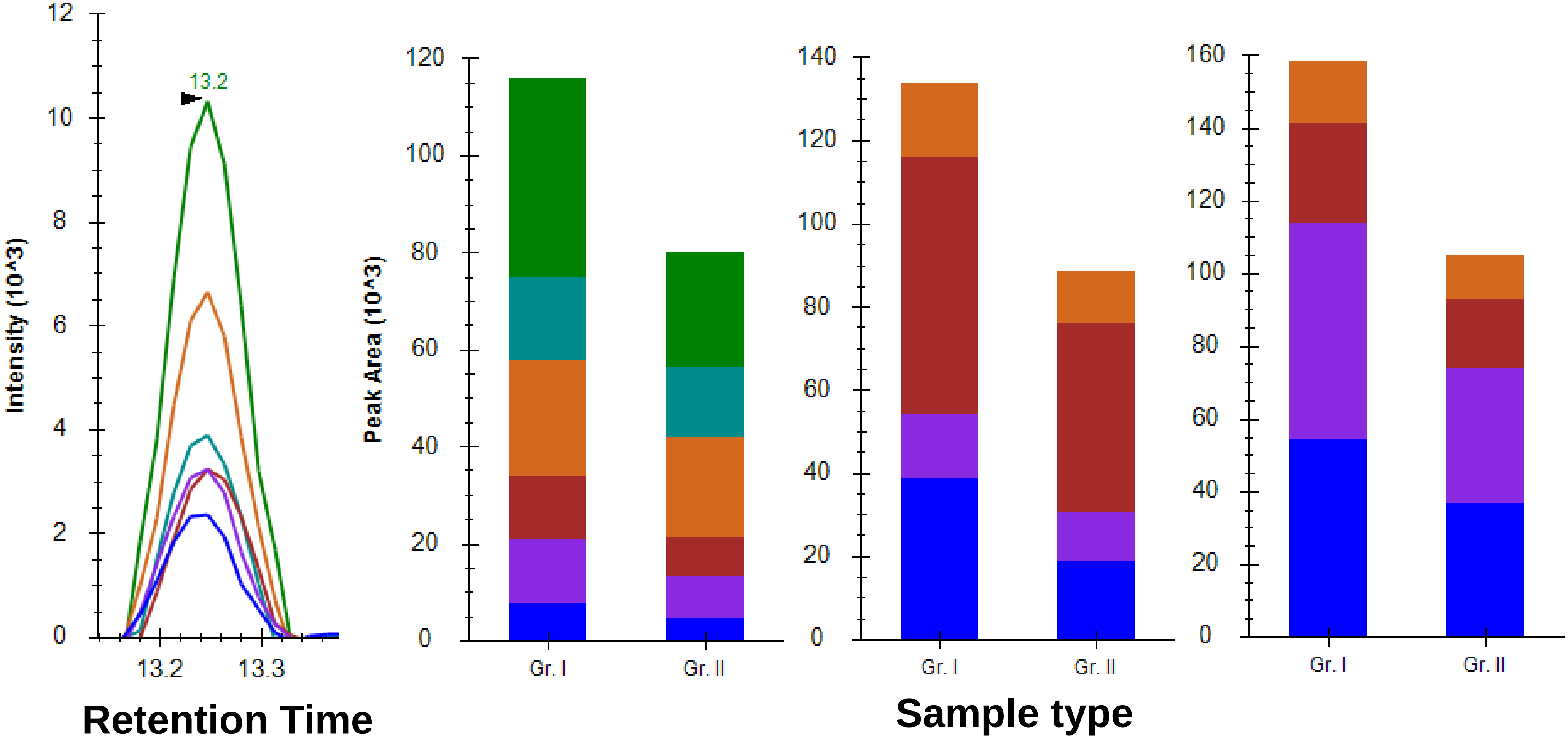

Representative MRM peak and bar graphs for SIAQYWLGCPAPGHL, FEDGVLDPDYPR and DVWGIEGPIDAAFTR of Vitronectin respectively, showing overexpression in Grade I as compared to Grade II.

Supplementary Figure 2A

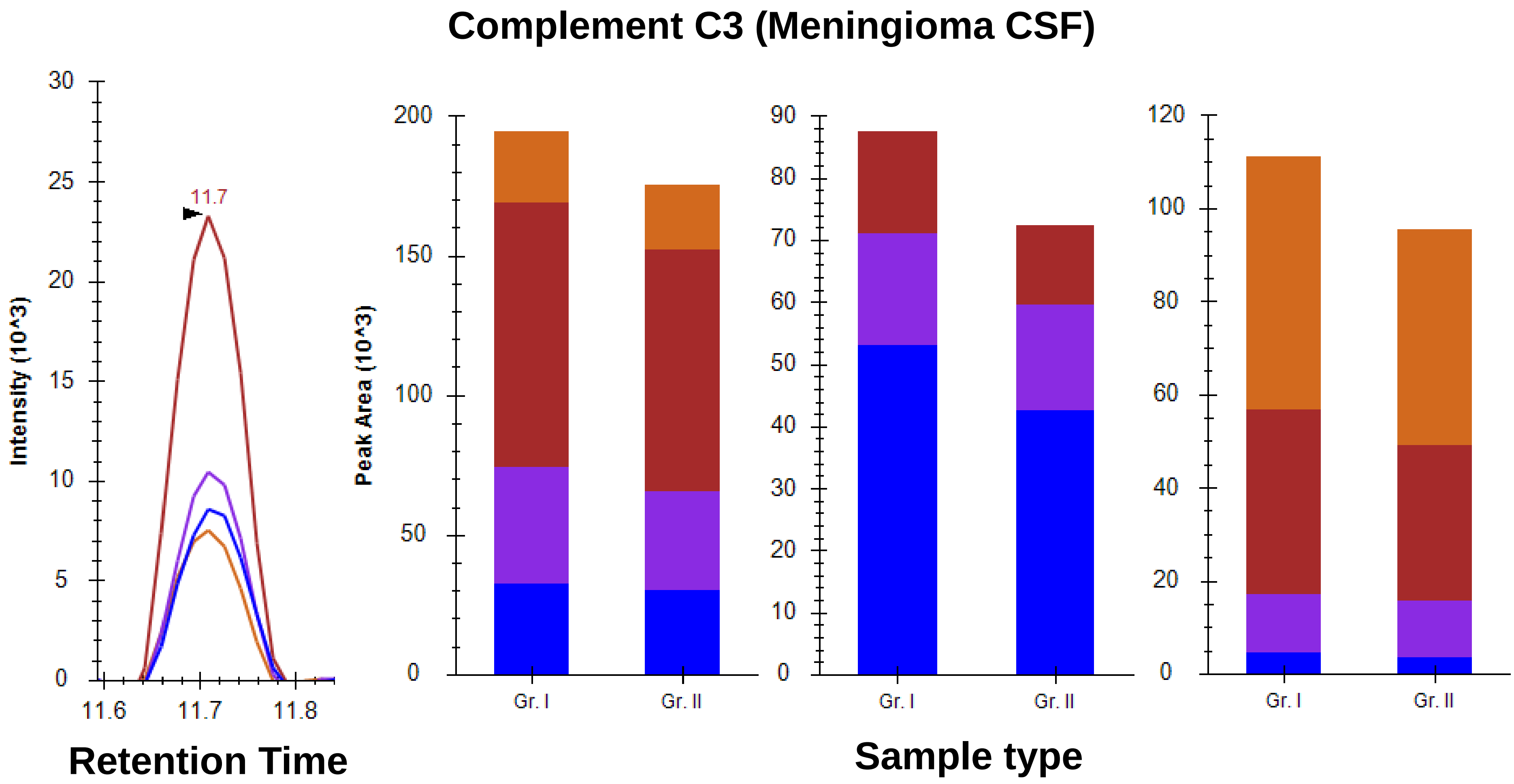

Representative MRM peak and bar graphs for LVAYYTLIGASGQR, TGLQEVEVK and SGSDEVQVGQQR of Complement C3 respectively, showing overexpression in Grade I as compared to Grade II.

Supplementary Figure 2A

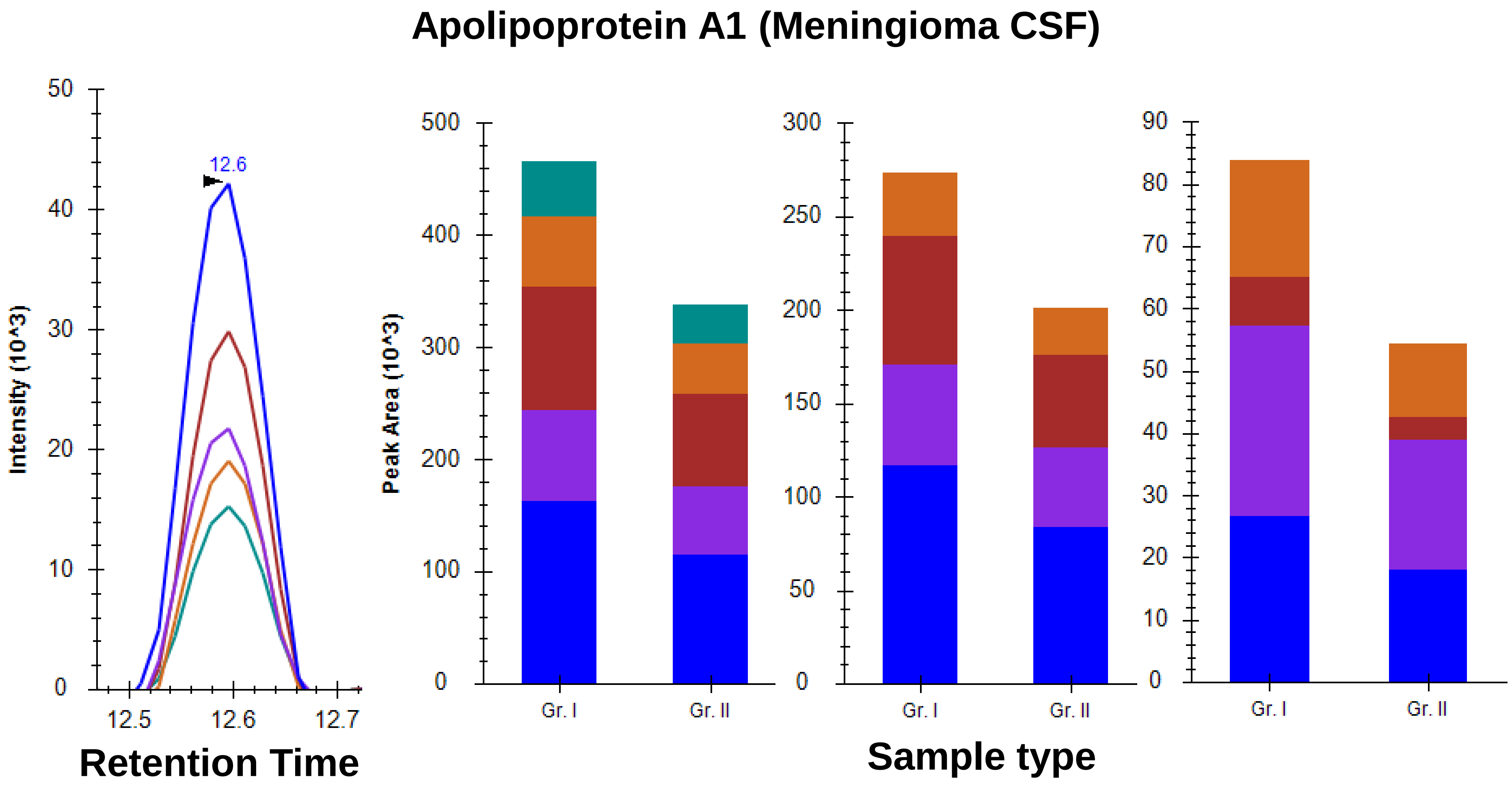

Representative MRM peak and bar graphs for LLDNWDSVTSTFSK, DYVSQFEGSALGK and ATEHLSTLSEK of Apolipoprotein A1 respectively, showing overexpression in Grade I as compared to Grade II.

Supplementary Figure 2B

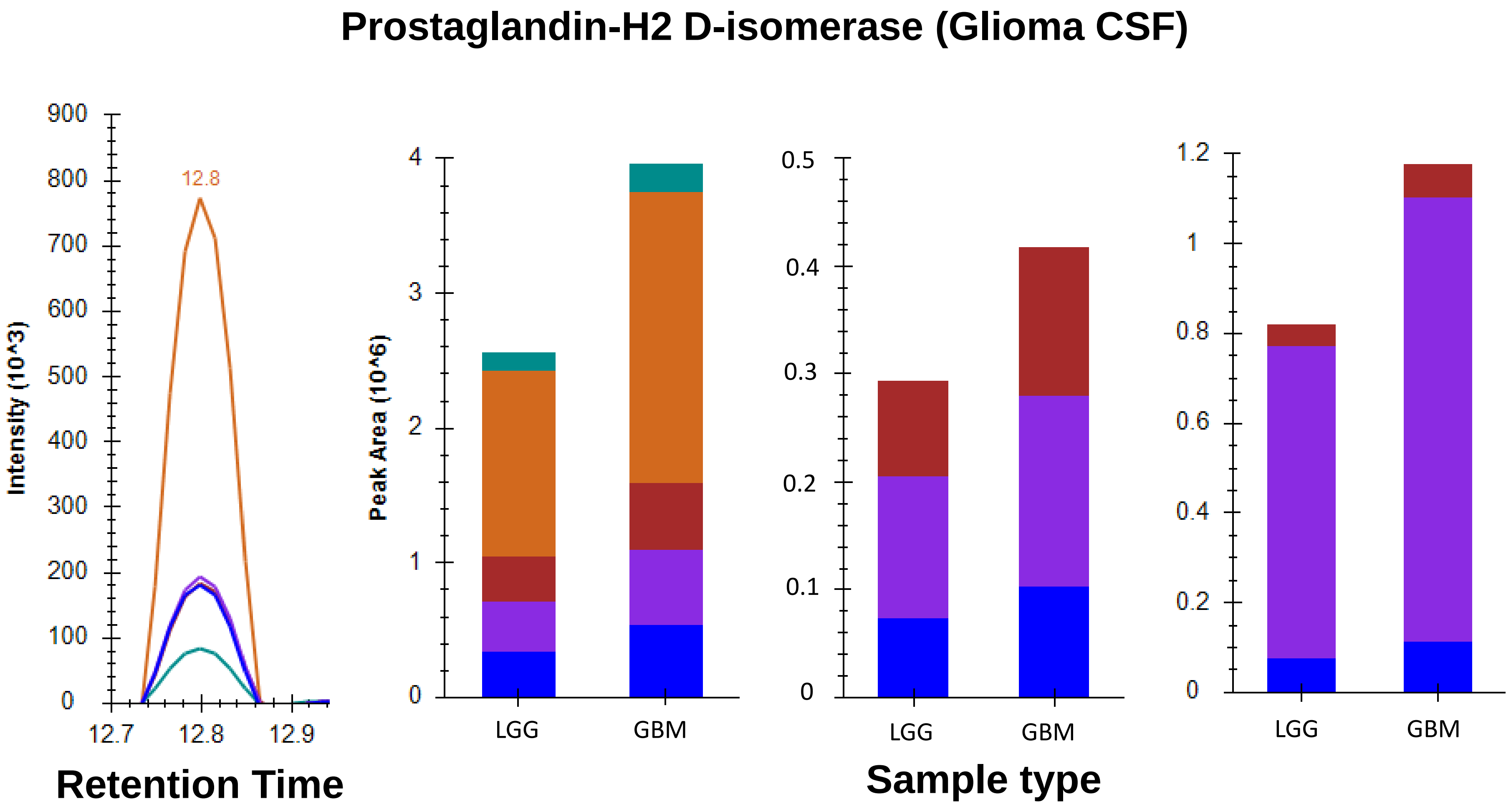

Representative MRM peak and bar graphs for TMLLQPAGSLGSYSYR, WFSAGLASNSSWLR and AQGFTEDTIVFLPQTDK of PTGDS respectively, showing overexpression in GBM as compared to LGG.

Supplementary Figure 2B

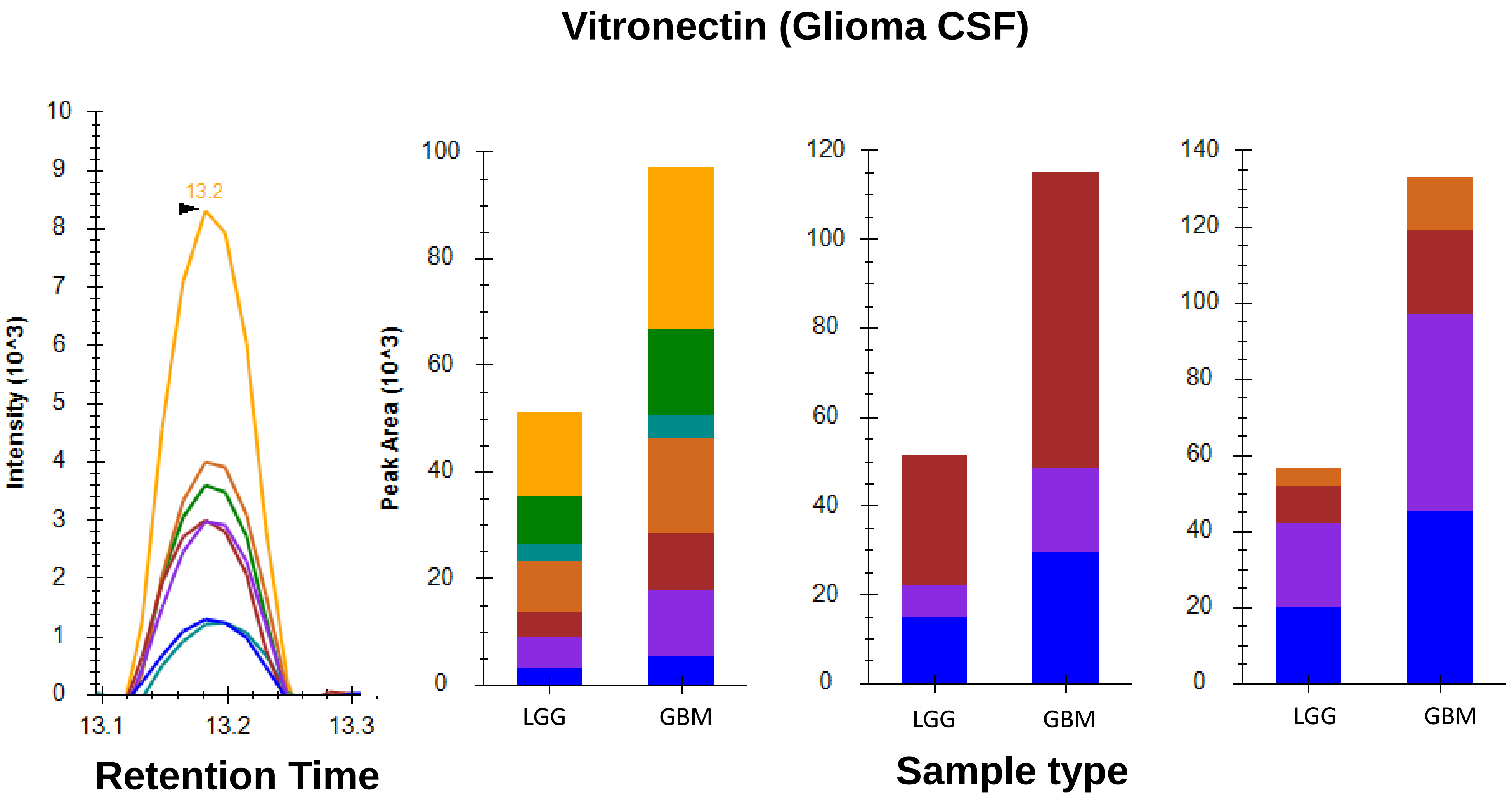

Representative MRM peak and bar graphs for SIAQYWLGCPAPGHL, FEDGVLDPDYPR and DVWGIEGPIDAAFTR of Vitronectin respectively, showing overexpression in GBM as compared to LGG.

Supplementary Figure 2B

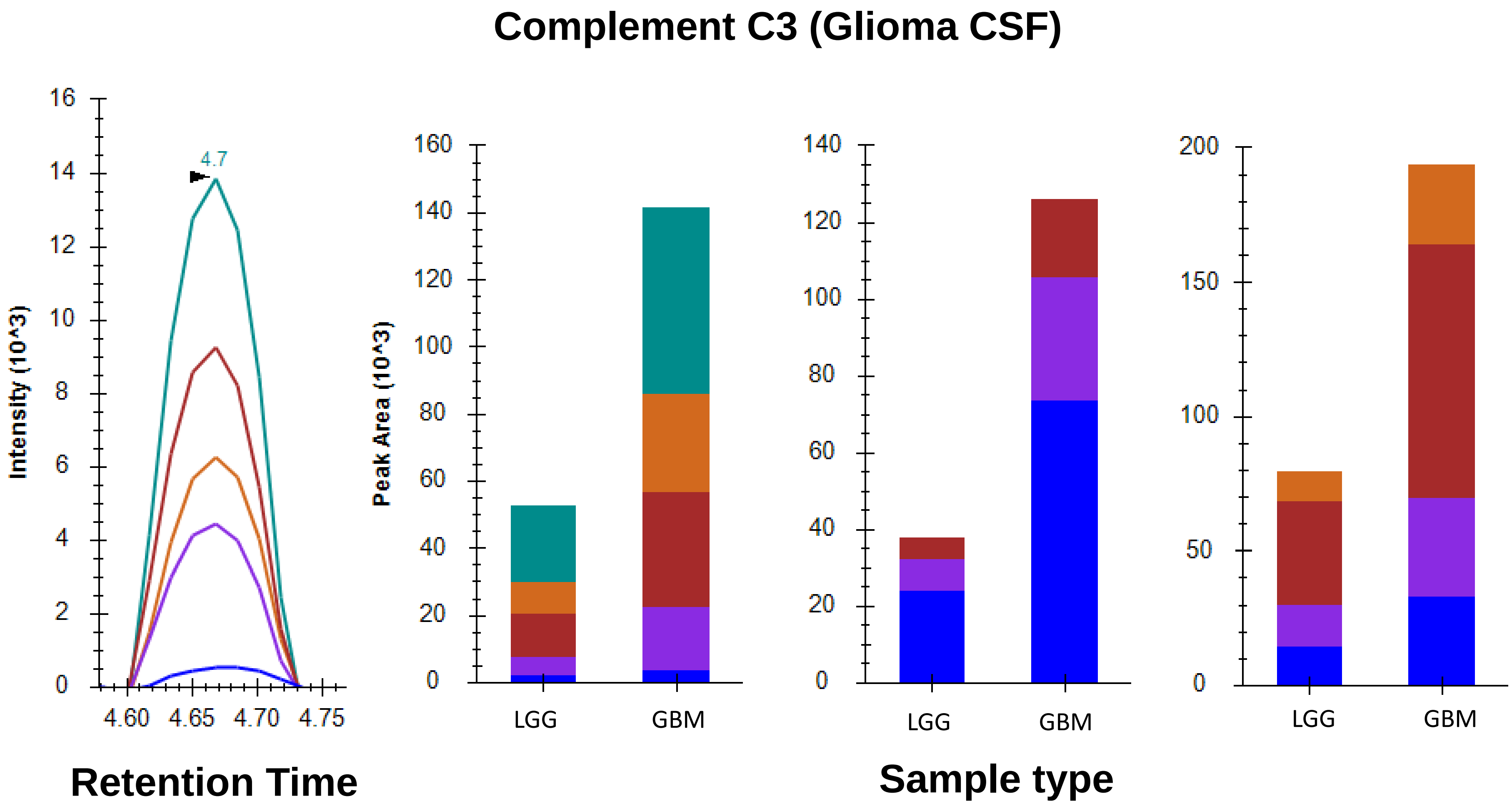

Representative MRM peak and bar graphs for SGSDEVQVGQQR, TGLQEVEVK and LVAYYTLIGASGQR of Complement C3 respectively, showing overexpression in GBM as compared to LGG.
