# Supplementary material for: Multiple Reaction Monitoring-Based Targeted Assays for the Validation of Protein Biomarkers in Brain Tumors
Source: Front Oncol. 2021 May 14;11:548243. doi: 10.3389/fonc.2021.548243 (PMC8162214; doi:10.3389/fonc.2021.548243)
Supplement: Supplementary file 3 [file Image_3.pdf]

Supplementary Figure 3A

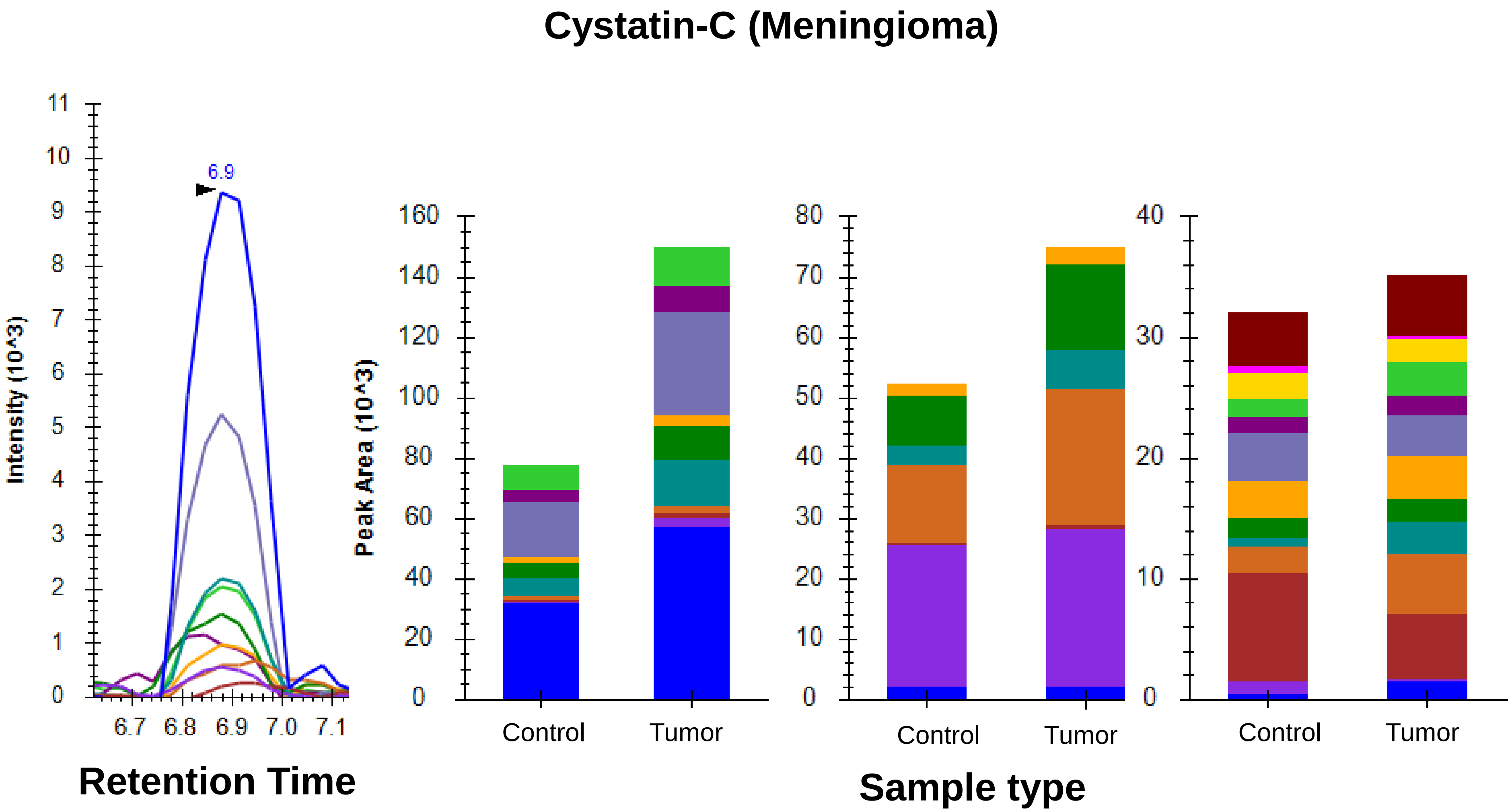

Representative MRM peak and bar graphs for LVGGPMDASVEEEGVR, QIVAGVNYFLDVELGR and TQPNLDNCPFHDQPHLK of Cystatin-C respectively, showing overexpression in tumor as compared to controls.

Supplementary Figure 3A

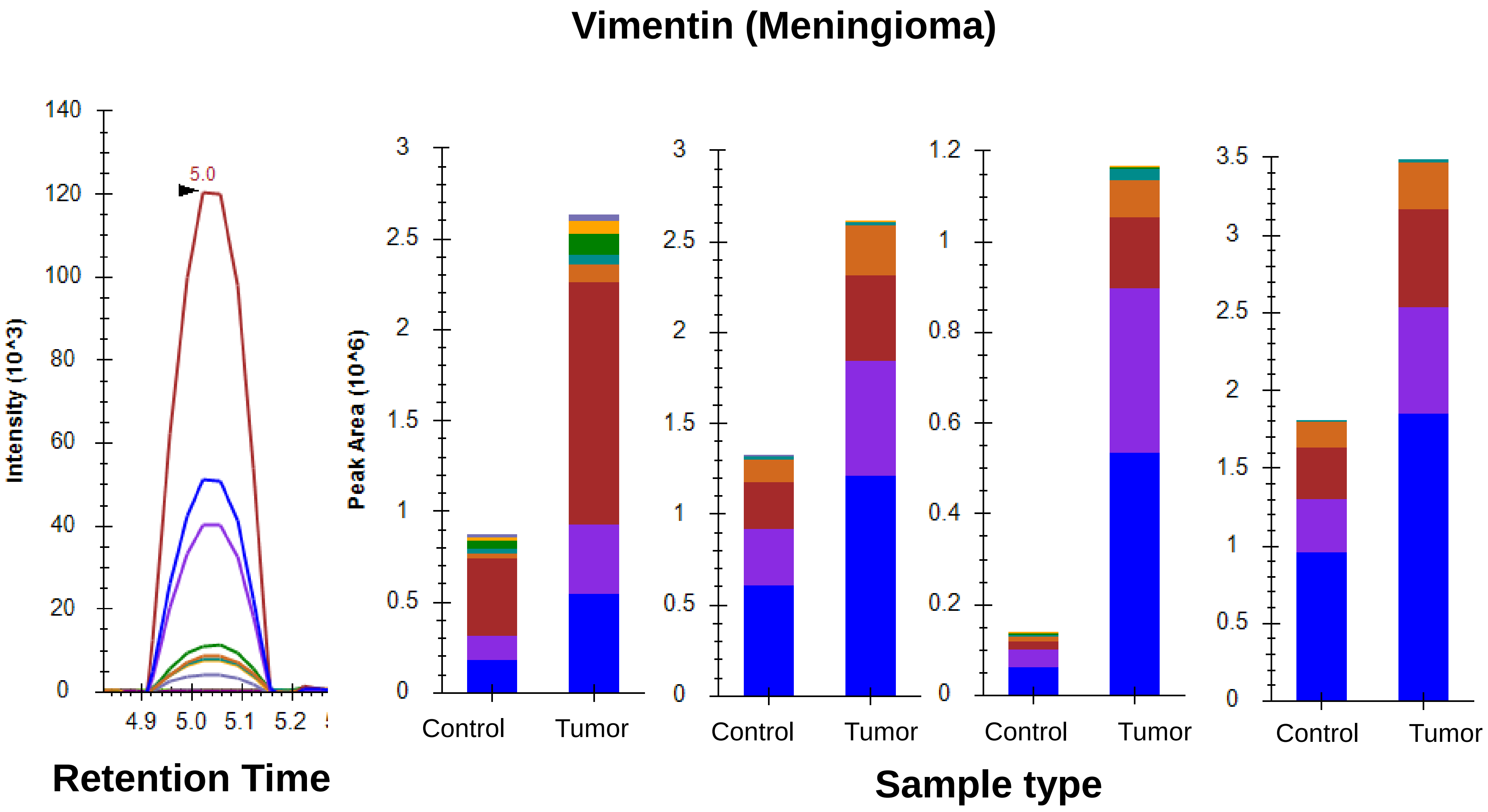

Representative MRM peak and bar graphs for SLYASSPGGVYATR, ILLAELEQLK, LGDLYEEEMR and FADLSEAANR of Vimentin respectively, showing overexpression in tumor as compared to controls.

Supplementary Figure 3B

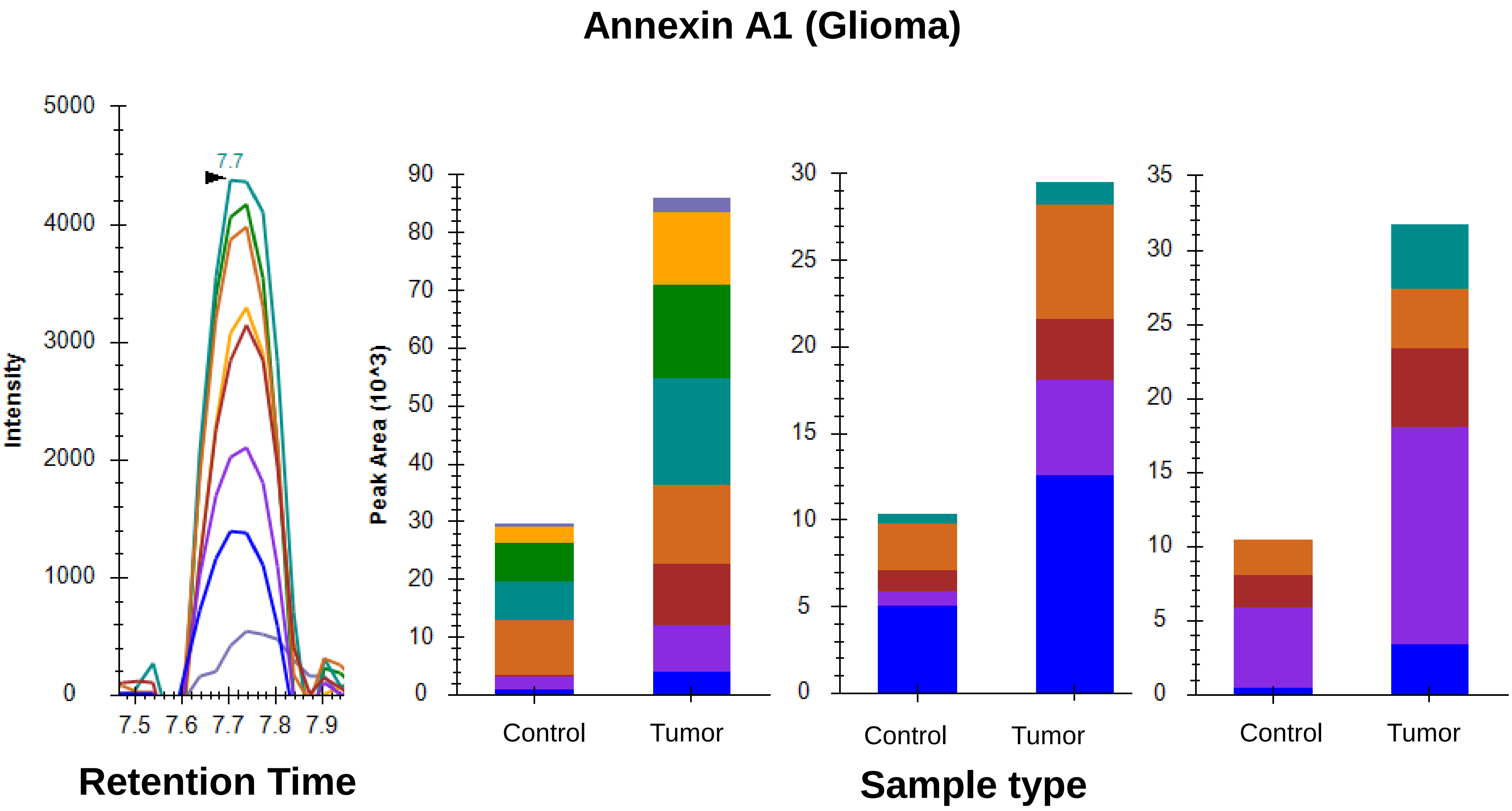

Representative MRM peak and bar graphs for GLGTDEDTLIEILASR, GVDEATIIDILTK and GTDVNVFNTILTTR of Annexin A1 respectively, showing overexpression in tumor as compared to controls.

Supplementary Figure 3B

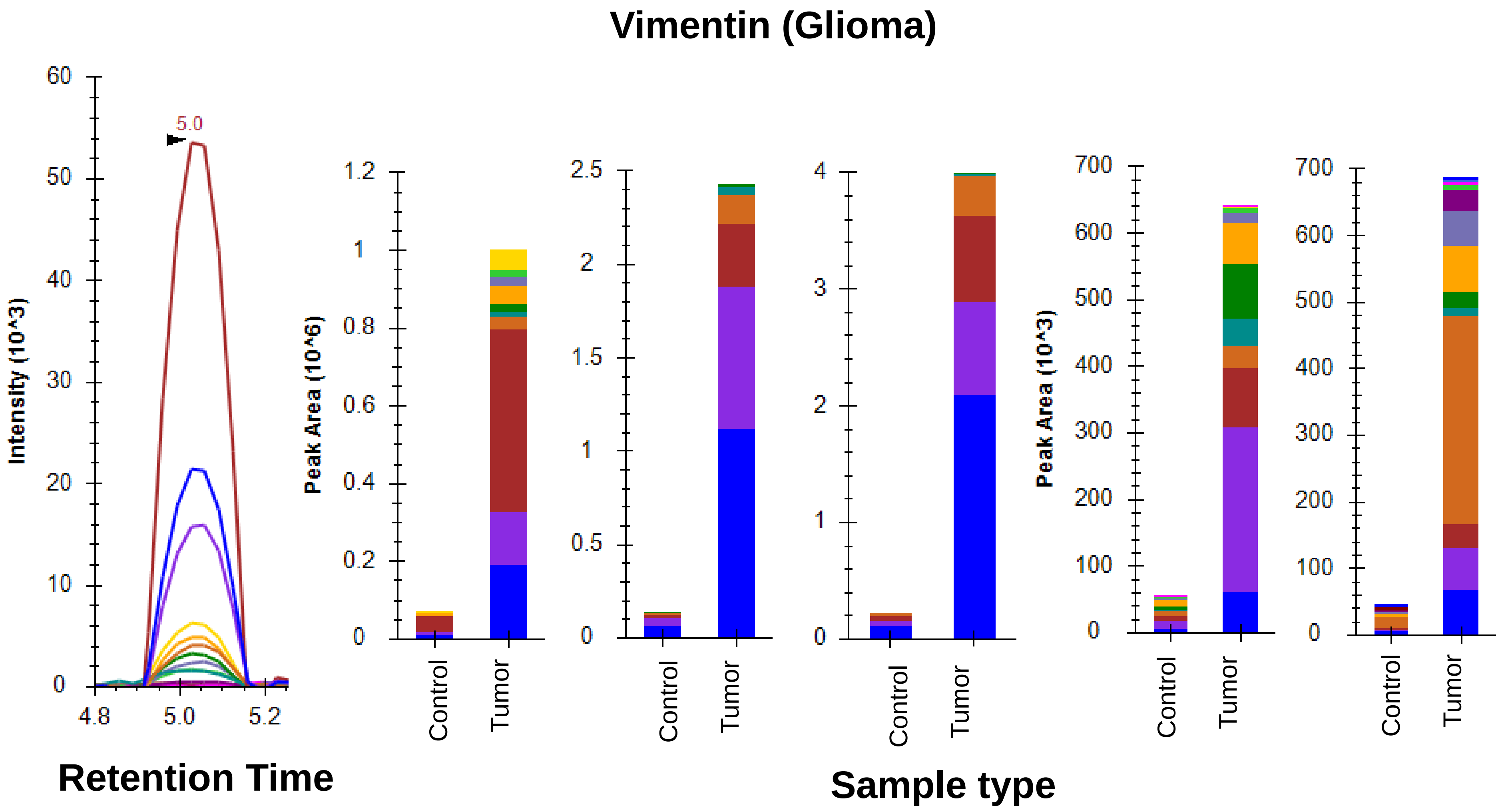

Representative MRM peak and bar graphs for SLYASSPGGVYATR, ILLAELEQLK, LGDLYEEEMR, FADLSEAANR, QVQSLTCEVDALK and ETNLDSLPLVDTHSK of Vimentin respectively, showing overexpression in tumor as compared to controls.
